# How do housing prices affect innovation and entrepreneurship? Evidence from China

**DOI:** 10.1371/journal.pone.0288199

**Published:** 2023-07-13

**Authors:** Jianshuang Fan, Dongtao Liu, Mingzhi Hu, Yipeng Zang

**Affiliations:** 1 Chinese Academy of Housing and Real Estate, School of Management, Zhejiang University of Technology, Hangzhou, China; 2 School of Management, Zhejiang University of Technology, Hangzhou, China; University of Southampton - Malaysia Campus, MALAYSIA

## Abstract

This paper analyzes how housing prices affect innovation and entrepreneurship. We construct a city-level panel dataset including 281 cities between 2009 and 2019 by merging housing price data from China Statistical Yearbook for Regional Economy with innovation and entrepreneurship data from Peking University Open Research Data Platform. Our results suggest that housing prices are positively associated with the vitality of innovation and entrepreneurship (VIE). The results remain consistent with a series of robustness checks. We also find that rising house prices promote VIE through the wealth effect and the siphon effect. Spatial effect analysis further shows that housing prices not only positively affect the VIE of local cities, but also positively affect the VIE of neighboring cities. These findings imply the necessity of curbing the excessive rise of housing prices and decoupling public services and benefits related to homeownership.

## 1. Introduction

Innovation and entrepreneurship are the keys to economic development [1]. The effectiveness of innovation-driven development directly determines a country’s future international competitiveness [2, 3]. Many studies have examined the determinants of innovation [4–7] and entrepreneurship [8–13]. The existing literature mainly focuses on the impact of housing prices on the behaviors or decisions of households and enterprises [14, 15], while less attention has been paid to the macro consequences of housing prices. This paper focuses on the association between housing prices and VIE. Theoretically, the relationship between housing prices and VIE is unclear. On the one hand, the wealth effect of housing prices eases the credit constraints that restrict innovative and entrepreneurial activities [16–18]. On the other hand, the appreciation of housing prices attracts investors and capital to the real estate market, crowding out new start-ups and innovative business ventures [19, 20].

This paper contributes to the literature in the following four aspects. First, it focuses on a comprehensive measure of innovation and entrepreneurship. Innovation and entrepreneurship have received widespread attention in the media and academic circles, while previous studies examine innovation and entrepreneurship separately. Second, it investigates the relationship between housing prices and VIE, which identifies a new source of diversity in innovation and entrepreneurship across regions. The existing literature either uses data at the provincial level or from broader regions to examine the relationship between housing prices and entrepreneurship or innovation [8, 21]. This study uses city-level data to provide a more accurate reference for urban policy formulation. This study constructs a city-level panel dataset between 2009 and 2019 by merging housing price data from China Statistical Yearbook for Regional Economy with VIE data from Peking University Open Research Data Platform. We find that housing prices are positively associated with VIE. This result is robust to a series of different model specifications. The findings of this study based on city-level data represent one of the most comprehensive examinations of how growth in housing prices is affecting entrepreneurship and innovation. Third, it unveils the mechanisms underlying why housing prices affect VIE. We show that rising house prices promote VIE through the wealth effect (rising house prices lead to an increase in asset value) and the siphon effect (high housing price of a city attracts talents for better market opportunities). Fourth, it considers the spatial spillover effect of housing prices and finds that a city’s housing prices affect the VIE of adjacent cities, which enriches our knowledge of the social and economic consequences of housing prices.

This study focuses on China which provides an important economic and policy context for the current research. China’s economic development has made rapid progress since the reform and opening up in 1978 [3]. Foreign capital and technology promote innovation and entrepreneurship [22]. With the transformation of Chinese economic system from planned economy to market economy, the private economy has developed unprecedentedly in China. With the rising cost of production and the waning competitive advantage of traditional industries, innovation and entrepreneurship have become increasingly important for the shift of China’s economic growth model from high-speed growth into high-quality development [23]. China implemented the Mass Entrepreneurship and Innovation (MEI) policy in 2015 to promote an innovation-driven development strategy. The state and local governments have attached great importance to the promotion effect of MEI policy on social and economic development. However, China has experienced a sustained and rapid increase in housing prices since the housing reform in 1994 [24]. According to data from the National Bureau of Statistics, from 1999 to 2020, nationwide housing prices had risen from 1843 yuan/m^2^ to 10448/m^2^. The soaring housing prices in China have aroused widespread concern [25–30]. The results of this paper are not only helpful for the Chinese government to formulate policies promoting the vitality of urban innovation and entrepreneurship but also have policy implications for the development of the real estate market.

The rest of the paper is structured as follows. Section 2 reviews the relevant literature. Section 3 introduces the data, variables, and methodologies. Section 4 presents the empirical results. The last section concludes the paper by discussing the implications of our findings.

## 2. Literature review

The impact of housing prices on the vitality of innovation and entrepreneurship is vague theoretically and therefore needs empirical investigation. On the one hand, housing prices have a positive impact on VIE. First, rising housing prices enhance the city’s innovation and entrepreneurship through the wealth effect. Housing is an important capital asset, and rising house prices lead to an increase in asset value [27]. The wealth effect of housing assets has been documented in numerous studies. A seminar work by Evans and Jovanovic [12] shows a significant positive correlation between household wealth and entrepreneurial engagement. Innovative and entrepreneurial activities are risky. Housing wealth enhances an individual’s risk-bearing ability and plays a positive role in innovation and entrepreneurship activities [31]. In addition, housing assets can be used as collateral. The accumulation of wealth from rising property prices helps ease the credit constraints that constrain innovation and entrepreneurship [17, 25, 32, 33]. For example, Chaney and Thesmar [34] analyze the impact of real estate value held by American companies on corporate investment, and their results show that the increase in the value of real estate has a significant promoting effect on enterprise investment. Li and Li [35] find that households owning mortgaged houses with full property rights are more likely to participate in entrepreneurship than households without full property rights, and this effect is more obvious in places where housing prices rise rapidly.

Second, rising housing prices enhance the city’s innovation and entrepreneurship vitality through the siphon effect. The Tiebout-Oates model predicts that urban housing prices depend on the quality of public services; in other words, public services should be capitalized into housing prices [36]. Housing prices, local public goods, and potential economic opportunities are linked [37]. A city’s high housing prices mean more job opportunities [38], better development prospects, and greater potential for wealth growth [18, 39], thus attracting more talents with high creativity and productivity. The gathering of a large number of talents is conducive to enhancing the vitality of urban innovation [39, 40].

On the other hand, housing prices have a negative impact on the vitality of innovation and entrepreneurship through the crowding-out effect. Investment opportunities in the real estate market squeeze investment in innovative and entrepreneurial activities. High returns on real estate caused by high housing prices encourage companies and investors to invest in real estate, crowding out innovative venture capital [16, 20, 41, 42]. For example, Chakraborty et al. [43] find that the appreciation of house prices has a crowding-out effect on venture capital and has a negative spillover effect on the real economy. Wang et al. [44] also find a negative correlation between housing prices and corporate investment. In addition, bank loans are an important source of investment in innovation and entrepreneurship activities. In the period of the rapid rise in housing prices, real estate-related investment has the characteristics of high returns and low risks. Therefore, banks are more willing to allocate loans to real estate industries during this period [45, 46]. Moreover, Chinese households have a tradition of valuing homeownership and owning a home even becomes a prerequisite for many young Chinese to get married. With a high ratio of house prices to incomes, many Chinese households that have bought homes have very high household debt. Debt pressures and a shortage of cash flow discourage households from venturing into risky entrepreneurial and innovative activities [31]. Furthermore, high housing prices will squeeze out innovative talents. The cost of urban housing has an important influence on the location choice of labor. Rising property prices push up the cost of living for residents [7], resulting in a migration of workers to cities with relatively low housing prices. The loss of human capital in cities with high housing prices has a negative impact on innovation and entrepreneurship activities [7, 18]. For example, Tsai [47] finds that labor migration behavior is largely driven by urban housing price differences. Chen et al. [24] show that high housing prices in China’s first-tier cities have a crowding-out effect on elites.

A city’s housing prices can be affected by its neighboring cities and also have a spillover effect on innovation and entrepreneurship [48, 49]. Considering the spatial interaction effects on regional housing prices [50, 51], the VIE of a city will be affected by the housing prices of adjacent cities. The effect comes from two aspects: first, the change of house prices in neighboring cities affects the house prices of local cities, thus affecting the VIE of local cities; second, the change of housing prices in neighboring cities causes the change of VIE in neighboring cities and thus affects the VIE of local cities through the spillover effect.

## 3. Data, variables, and methodologies

### 3.1 Data

To examine the relationship between housing prices and VIE, we construct a city-level panel dataset between 2009 and 2019 by merging housing price data from China Statistical Yearbook for Regional Economy with VIE data from Peking University Open Research Data Platform. Data of mediating variables and control variables come from China Statistical Yearbook for Regional Economy and China Urban Statistical Yearbook. We focus on data obtained after the 2007–2008 financial crisis and before the first COVID-19 pandemic wave that hit the world economy between the winter of 2019 and the spring of 2020. 281 cities are included in the study.

### 3.2 Variables

#### 3.2.1 Dependent variable: VIE

We use the Index of Regional Innovation and Entrepreneurship in China (IRIEC) from the Peking University Open Research Data Platform. It is a good proxy for VIE for three reasons. First, IRIEC covers all industries, including especially small and micro enterprises that are considered as the backbone of innovation and entrepreneurship. Second, the index is calculated based on nearly 50 million records of a variety of indicators, which can reflect the vitality of innovation and entrepreneurship. Third, the index is constructed based on a multi-dimensional comprehensive evaluation system that covers multi-dimensional indicators. Specifically, IRIEC is the weighted average of five indicators: number of new enterprises (20%), foreign capital investments (15%), venture capital investments (25%), number of patents granted (25%), and number of trademark registration (15%).

#### 3.2.2 Independent variable: Housing prices

Housing prices are measured as the ratio of the one-year lagged total sales of commercial housing to the one-year lagged sales area [9, 52]. Housing prices are deflated by CPI to convert into real values. Finally, we take the log of housing prices to reduce heteroscedasticity.

#### 3.2.3 Mediating variable: Consumption willingness

We use the log value of consumption expenditure per capita to measure the consumption willingness. With the increase of market demand for the diversity of products, enterprises are encouraged to develop new technologies and products. Kerr et al. [53] find that the relationship between housing prices and entrepreneurship mainly depends on the level of local market demand. Rising housing prices promote household consumption [15], and thus have a positive effect on VIE [19].

#### 3.2.4 Mediating variable: Consumption ability

We use the log value of the average wage to measure consumption ability. Rising housing prices not only increase the production and operation costs of enterprises but also increase the wage which undermines economic competitiveness [54]. The increase in wages in accordance with rising housing prices enhances household consumption and market demand for innovative products [45]. Besides, it increases the attractiveness of people with high education and skills, which is conducive to promoting innovation and applied technological research.

We follow previous studies and choose the following eight control variables: (1) *City size*. The literature suggests a significant and positive correlation between population and innovation [10, 55]. We use the log value of the total population at the end of the year to measure city size. (2) *Human capital*. A large number of talents is a necessary condition to enhance the vitality of urban innovation [39, 40]. We use the log value of the number of full-time teachers at colleges and universities to measure the level of human capital. (3) *Economic growth*. Both economic growth and industrial structure are important factors affecting urban innovation [56]. We use the log value of per capita GDP to measure economic growth. Per capita GDP is deflated by CPI to convert into real values. (4) *Industrial structure*. Industrial structure has a significant impact on innovation [57]. We use the ratio of the value-added of the tertiary industry to the value-added of the secondary industry to measure industrial structure. (5) *Trade openness*. We use the log value of imports and exports per capita as a proxy for trade openness, which is suggested to be an important factor of innovation and entrepreneurship [58, 59]. (6) *Infrastructure*. Good infrastructure attracts workers and businesses, thus helping to improve the vitality of innovation and entrepreneurship [60]. We use the log value of the number of hospital beds per 10,000 people to measure infrastructure. (7) *Research and development*. The intensity of government investment in science and technology has a significant and positive impact on urban innovation [6]. We use the ratio of government expenditures on science and technology to public expenditures to measure the government’s inputs on research and development. (8) *Informatization*. We use the log value of the number of Internet users to measure informatization, which is also associated with innovative and entrepreneurial activities [61]. Table 1 presents the definitions and summary statistics of variables.

**Table 1:** Definitions and summary statistics of variables.

Variables	Definitions	Mean	Min.	Max.
VIE	Log value of IRIEC	3.718	0.311	4.605
Commercial housing prices	Log value of the ratio of the one-year lagged total sales of commercial housing to the one-year lagged sales area	7.930	6.528	10.472
Consumption ability	Log value of average wage level of employees	10.752	8.509	11.772
Consumption willingness	Log value of consumption expenditure per capita	4.178	3.822	4.654
City size	Log value of the total population at the end of the year	5.856	2.970	7.313
Human capital	Log value of the number of full-time teachers at colleges and universities	7.553	3.258	11.117
Economic growth	Log value of GDP per capita	10.141	8.046	12.688
Industrial structure	Ratio of the value-added of the tertiary industry to the value-added of the secondary industry	0.937	0.109	4.932
Trade openness	Log value of imports and exports per capita	7.879	0.000	13.891
Infrastructure	Log value of the number of hospital beds per 10,000 people	3.698	2.357	4.925
Research and development	Ratio of government expenditures on science and technology to public expenditures	0.015	0.001	0.207
Informatization	Log value of the number of Internet users	3.997	0.875	6.907

Note: Housing price data come from China Statistical Yearbook for Regional Economy. VIE data come from Peking University Open Research Data Platform. Data of other variables come from China Statistical Yearbook for Regional Economy and China Urban Statistical Yearbook.

**Fig 1:**
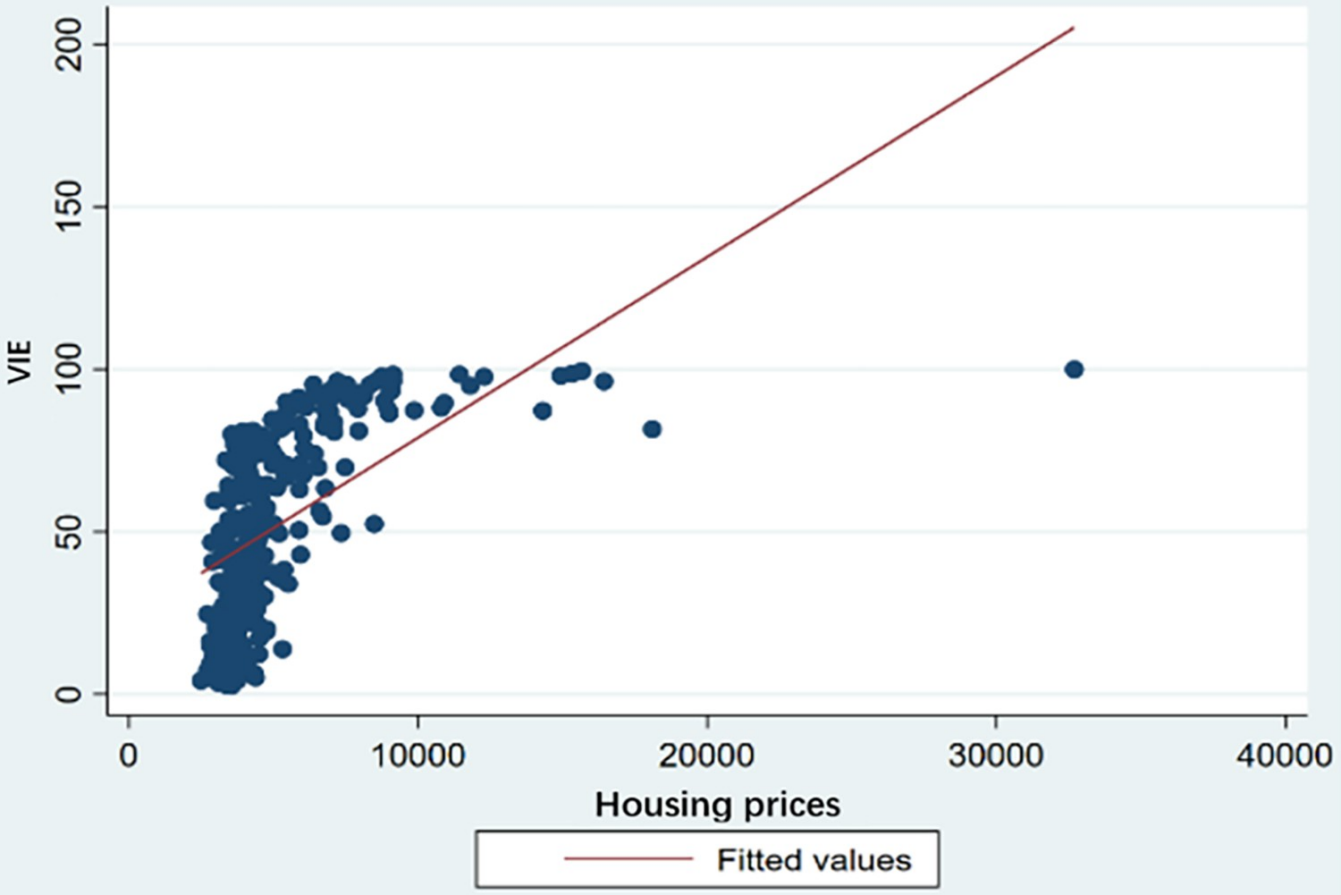
Correlation between housing prices and VIE.

Fig 1 shows a scatter graph between housing prices and VIE measured at the city level. Again, the figure shows that housing prices is positively correlated with VIE. However, analyses that control for other factors critical for innovation and entrepreneurship are needed.

### 3.3 Econometric regression models

#### 3.3.1 Baseline regression

To test how housing price affects VIE, we conduct the following baseline regression model:

$${VIE}_{it}=\alpha_{0}+\alpha_{1}{Housing\;prices}_{it-1}+{\beta X}_{it}+u_{i}+u_{t}+\varepsilon_{it}$$ (1)

where *VIE*_*it*_ represents the log value of VIE in city *i* and year *t*. *Housing Price*_*it*−1_ represents the log value of housing prices in city *i* and year *t-*1. *X*_*it*_ represents a set of control variables as described in Table 1. *α*_0_ is the constant. *α*_1_ is the coefficient of interest that measures the impact of housing prices on VIE. *β* is the coefficient matrix that measures the impacts of control variables on VIE. *μ*_*i*_ and *μ*_*t*_ represent city fixed effect and year fixed effect. *ε*_*it*_ is the error term.

#### 3.3.2 Mediating regression

We further examine the mediating effect of consumption willingness and consumption ability by using the models proposed by Baron and Kenny [62], which are set as follows:

$${Mediator}_{it}=\beta_{0}+\beta_{1}{Housing\;prices}_{it-1}+{\gamma X}_{it}+u_{i}+u_{t}+\varepsilon_{it}$$ (2)

$${VIE}_{it}=c_{0}+c_{1}{Housing\;prices}_{it-1}+c_{2}{Mediator}_{it}+{\beta X}_{it}+u_{i}+u_{t}+\varepsilon_{it}$$ (3)

where *Mediator*_*it*_ represents the mediating variable that takes two outcomes: consumption willingness and consumption ability. *β*_1_ measures the impact of housing prices on the mediating variable. *c*_1_ represents the impact of housing prices on VIE after controlling for the mediating effect. *c*_2_ represents the impact of the mediating variable on VIE. Other variables are defined the same as those in Eq (1).

#### 3.3.3 Spatial econometric regression

Housing prices, innovation, and entrepreneurship may have spatial spillover effects [48, 49, 63]. We further examine the potential spatial spillover effect of housing prices on VIE with a series of spatial econometric regressions, including spatial autoregressive model (SAR), spatial autocorrelation model (SAC), and spatial error model (SEM). The SAR is set as follows:

$${VIE}_{it}=\rho W\times {VIE}_{it}+\alpha {Housing\;prices}_{it-1}+{\beta X}_{it}+u_{i}+u_{t}+\varepsilon_{it}$$ (4)

where *ρ* is spatial autoregressive coefficient. *W* is spatial weight matrix. We use two types of weight matrices: distance weight matrix and inverse distance weight matrix.

The SAC is set as follows:

$${VIE}_{it}=\rho W\times {VIE}_{it}+\alpha {Housing\;prices}_{it-1}+\beta X_{it}+u_{i}+u_{t}+\xi_{it}$$ (5)

$$\xi_{it}=\lambda W\xi_{it}+\varepsilon_{it}$$ (6)

where *λ* is a spatial error coefficient. The SEM is set as follows:

$${VIE}_{it}=\alpha {Housing\;prices}_{it-1}+{\beta X}_{it}+u_{i}+u_{t}+\zeta_{it}$$ (7)

$$\zeta_{it}=\lambda W\zeta_{it}+\varepsilon_{it}$$ (8)

## 4. Empirical results

Table 2 reports the variance inflation factor (VIF) for the explanatory variables. We can see that the maximum VIF value is 4.33, which is far less than the threshold value of 10. Therefore, the threat from multicollinearity is limited.

**Table 2:** The variance inflation factor of each explanatory variable.

	Housing prices	City size	Human capital	Economic growth	Industrial structure	Trade openness	Infrastructure	Research and development	Informatization
VIF	3.30	4.09	2.85	3.82	1.38	2.73	2.58	1.72	4.33

### 4.1 Baseline results

The random effect estimator is more efficient when unobservables are uncorrelated with covariate *X*_*it*_, the fixed effect estimator is more efficient when unobservables are correlated with covariate *X*_*it*_. We use the Hausman test to examine whether the fixed-effects or random-effects model is suitable for panel data regression. Results from the Hausman test show that the random-effects mode is rejected at the 1% of statistical significance (Chi-squared = 360.48), and thus the fixed-effects model is suitable in comparison with the random-effects model. We present the baseline results from Eq (1) based fixed-effects model in Table 3.

**Table 3:** Baseline regression results.

	(1)	(2)
	VIE	VIE
Housing prices	0.268***	0.183***
	(5.02)	(3.46)
City size		0.544***
		(4.32)
Economic growth		0.205***
		(4.00)
Trade openness		0.044***
		(2.93)
Human capital		0.133***
		(4.80)
Infrastructure		0.241***
		(3.79)
Industrial structure		-0.094***
		(-2.82)
Informatization		0.076***
		(4.07)
Research and development		1.406*
		(1.82)
Constant	1.519***	-5.685***
	(3.47)	(-5.30)
R^2^	0.36	0.72
Year dummies	Yes	Yes
City dummies	281	281
Observations	2984	2984

Note: The dependent variable in this table is the log value of VIE. t-values are reported in parentheses.

*, **, and *** indicate significance at the 10%, 5%, and 1% levels, respectively.

To ensure the sensitivity of the relationship between housing prices and VIE, we first only control for housing prices and city and year fixed effects, as shown in column (1), and further add a set of control variables, as shown in column (2). The coefficient of *Housing prices* is positive and remains statistically significant at the 1% level in columns (1) and (2) of Table 3. These findings suggest that rising housing prices increase the validity of innovation and entrepreneurship. As discussed above, housing prices have not only the positive wealth effect and the siphonic effect on VIE, but also the negative crowding-out effect. Our results suggest that the positive effects of housing prices dominate, which is consistent with the findings of Hu & Chen [21] and Lin et al. [18].

### 4.2 Robustness checks

#### 4.2.1 Instrument variable approach

Although we have used the one-year lagged housing prices and controlled for many city characteristics, concerns on the endogeneity issue of housing prices still exist. There are two main reasons for the endogeneity. First, urban innovation and entrepreneurship activities may promote the income growth of enterprises and residents by increasing the added value of products, thus increasing the demand of the real estate market and driving up the local housing price. In other words, there may be a reverse causality between housing prices and innovation and entrepreneurship. Secondly, there are many factors affecting the vitality of urban innovation and entrepreneurship, and the empirical model cannot control all the relevant variables. We try to ease this concern using the instrument variable (IV) approach. The endogeneity issue of housing prices would be ideally addressed with an exogenous variable that affects VIE only directly through housing prices. We choose the one-year lagged value of the per capita construction land area as the IV for housing prices in this paper. The IV satisfies the conditions of instrument relevance and instrument exogeneity. On the one hand, construction land supply areas and housing prices are closely related [64]. Many studies have shown that land supply restrictions are the main factor leading to high housing prices [65]. The reduction of land supply leads to a rapid rise in housing prices. On the other hand, land supply is not directly correlated to innovation and entrepreneurship.

Results from the IV approach are reported in column (1) of Table 4. The F-statistic in the first stage regression, as reported in column (1) of Table 4, is 19.83 and statistically significant at the 1% level, supporting the validity of the IV. The coefficient of *Housing prices* remains positive and statistically significant at the 1% level. This finding suggests that the baseline results are robust to the endogeneity issue of housing prices.

**Table 4:** Robustness results.

	(1)	(2)	(3)	(4)
	VIE	Entrepreneurial vitality	Innovative vitality	VIE
Housing prices	2.031***	0.262***	0.323***	
	(2.74)	(8.30)	(6.39)	
PIR				0.434**
				(1.99)
Control variables	Yes	Yes	Yes	Yes
Year dummies	Yes	Yes	Yes	Yes
City dummies	Yes	Yes	Yes	Yes
F-statistics in the first stage	19.83***			

Note: The dependent variable in columns (1) and (4) is the log value of VIE. The dependent variable in column (2) is the log value of entrepreneurial vitality. The dependent variable in columns (3) is the log value of innovative vitality. t-values are reported in parentheses. *, **, and *** indicate significance at the 10%, 5%, and 1% levels, respectively.

#### 4.2.2 Alternative definition of key variables

In addition, we measure the vitality of innovation and entrepreneurship with two alternative variables. One is the log value of the number of newly established enterprises, which is used to measure the entrepreneurial vitality. Another is the weighted average value of the number of invention patents (50%), the number of utility patents (30%), and the number of design patents (20%), following the method of Bai & Jiang [66]. We take the log of the weighted value to measure the innovative vitality. The results from the baseline model by changing the dependent variable with entrepreneurial vitality and innovative vitality are reported in columns (2) and (3) of Table 4, respectively. Again, we can see that the results remain robust by using alternative dependent variables.

Similar to Chen & Hu [27] and Li & Wu [19], we use the ratio of housing prices to income (PIR) as an alternative measure of housing prices. The results with the PIR as the independent variable are reported in column (4) of Table 4. The coefficient of PIR equals 0.434, which is statistically significant at the 5% level.

### 4.3 Mediating analyses

#### 4.3.1 Consumption willingness

Results from Eq (2) with the consumption willingness as the mediating variable are reported in columns (1) of Table 5. The estimated coefficient of *Housing prices* is 0.058 and statistically significant at the 1% significance level, which suggests a positive effect of housing prices on households’ consumption willingness. Column (2) of Table 5 shows the results from Eq (3) with consumption willingness as the mediating variable. We observe a positive and significant impact of consumption willingness on the vitality of innovation and entrepreneurship. Besides, the coefficient of *Housing prices* remains positive and significant. These findings suggest a partial mediating effect of consumption willingness on VIE.

**Table 5:** The mediating effect of consumption willingness and consumption ability.

	(1)	(2)	(3)	(4)
	Consumption willingness	VIE	Consumption ability	VIE
Housing prices	0.058***	0.164***	0.104***	0.168***
	(3.24)	(3.07)	(6.49)	(3.15)
Consumption willingness		0.236***		
		(4.09)		
Consumption ability				0.144**
				(2.28)
Control variables	Yes	Yes	Yes	Yes
City dummies	Yes	Yes	Yes	Yes
Year dummies	Yes	Yes	Yes	Yes
R^2^	0.60	0.73	0.51	0.72
F-statistics	1609.69***	9.58***	1576.29***	9.07***
Observations	2968	2968	2984	2984

Note: The dependent variable in columns (1) is the log value of consumption willingness. The dependent variable in columns (2) and (4) is the log value of VIE. The dependent variable in columns (3) is the log value of consumption ability. t-values are reported in parentheses. *, **, and *** indicate significance at the 10%, 5%, and 1% levels, respectively.

#### 4.3.2 Consumption ability

We now check the mediating role of consumption ability. We report the results from Eq (2) with the consumption ability as the mediating variable in column (3) of Table 6. The estimated coefficient of *Housing prices* is 0.104 and statistically significant at the 1% significance level, suggesting that rising housing prices increase the consumption ability of households. This finding is consistent with previous research [67]. In column (4) of Table 6, we report the results from the baseline model by adding the log value of consumption ability. The estimated coefficient of *Housing prices* is 0.168 and statistically significant at the 1% significance level. These findings indicate that there is a partial mediating effect of consumption ability on VIE.

**Table 6:** Empirical test of spatial spillover effect.

	SAR		SAC		SEM	
	(1)	(2)	(3)	(4)	(5)	(6)
	Distance weight matrix	Inverse distance weight matrix	Distance weight matrix	Inverse distance weight matrix	Distance weight matrix	Inverse distance weight matrix
Housing prices	0.187***	0.173***	0.128***	0.174***	0.197***	0.183***
	(3.83)	(3.56)	(3.11)	(3.53)	(3.94)	(3.67)
R^2^	0.73	0.74	0.71	0.74	0.72	0.72
*ρ*	0.124***	0.610***	0.496***	0.514***		
*λ*			-0.47***	0.289*	0.115***	0.576***
Observations	3091	3091	3091	3091	3091	3091
Control variables	Yes	Yes	Yes	Yes	Yes	Yes
City dummies	Yes	Yes	Yes	Yes	Yes	Yes
Time dummies	Yes	Yes	Yes	Yes	Yes	Yes

Note: The dependent variable in columns (1) is the log value of consumption willingness. The dependent variable in columns (2) and (4) is the log value of VIE. The dependent variable in columns (3) is the log value of consumption ability. t-values are reported in parentheses. *, **, and *** indicate significance at the 10%, 5%, and 1% levels, respectively.

These findings provide supportive evidence that rising house prices can enhance the vitality of innovation and entrepreneurship through the wealth effect and the siphon effect.

### 4.4 Spatial econometric results

Table 6 reports the results from spatial econometric models. We use three different spatial econometric regressions (namely SAR, SAC, and SEM) as robustness checks. Columns (1) and (2) of Table 6 show results from the SAR, columns (2) and (3) show results from the SAC, and columns (4) and (5) show results from the SEM. Results from spatial econometric models may be sensitive to the weight matrix selected. To ensure the reliability of estimation results, we use two types of weight matrices. We choose the distance weight matrix in columns (1), (3) and (5), and the inverse distance weight matrix in columns (2), (4) and (6).

Results in columns (1) to (4) of Table 6 show that the spatial autoregressive coefficients (*ρ*) are positive and significant, indicating significant spatial spillover effects of VIE. A significant value of spatial error coefficient (*λ*) is also observed in columns (3) to (6) of Table 6, which also suggests the existence of the spatial spillover effect of VIE. The estimated coefficients of *housing prices* remain positive and statistically significant at the 1% level throughout columns (1) to (6). These findings suggest that the positive relationship between housing prices and VIE holds after controlling for spatial autocorrelations.

According to the method of Elhorst [68], we further decompose the total effect of housing prices on VIE into the direct effect and the indirect effect. The direct effect measures the impact of housing prices on VIE without any spatial spillover effect, and the indirect effect measures the change of VIE caused by the spatial spillover effect. The decomposition results of the impact of housing prices on VIE are shown in Table 7. Columns (1) and (2) shows a positive and significant direct and indirect effect of housing prices on VIE based on the SAR. The spatial spillover effect accounts for 12.09% of the total effect (i.e., 0.026 / (0.026 + 0.189) = 12.09%). Similar findings are suggested based on the SAC, although a large proportion of direct effect of housing prices on VIE is found (i.e., 0.118 / (0.118 + 0.139) = 45.91%).

**Table 7:** Direct and indirect effects of housing prices on VIE.

	SAR		SAC	
	(1)	(2)	(3)	(4)
	Direct effect	Indirect effect	Direct effect	Indirect effect
Housing prices	0.189***	0.026***	0.139***	0.118***
	(3.77)	(3.00)	(3.08)	(2.97)
Control variables	Yes	Yes	Yes	Yes
City dummies	Yes	Yes	Yes	Yes
Year dummies	Yes	Yes	Yes	Yes

Note: The dependent variable in this table is the log value of VIE. t-values are reported in parentheses. *, **, and *** indicate significance at the 10%, 5%, and 1% levels, respectively.

As a robustness check for the spatial econometric regression results, we also change the dependent variable in the spatial econometric models with *Innovation vitality* and *Entrepreneurial vitality*, separately. Table 8 reports the results by using alternative definitions of innovation and entrepreneurship. We only report the results using the distance weight matrix since our results are quite similar when we choose the inverse distance weight matrix. The estimated coefficient of *Housing prices* throughout columns (1) to (6) of Table 8 remains positive and significant, providing supportive evidence to our baseline results.

**Table 8:** Robustness checks.

	(1)	(2)	(3)	(4)	(5)	(6)
	SAR	SAC	SEM	SAR	SAC	SEM
	Innovation vitality			Entrepreneurial vitality		
Housing prices	0.25***	0.20***	0.24***	0.15***	0.12***	0.09***
	(5.59)	(4.46)	(5.05)	(4.70)	(3.78)	(2.59)
Control variable	Yes	Yes	Yes	Yes	Yes	Yes
City dummies	Yes	Yes	Yes	Yes	Yes	Yes
Year dummies	Yes	Yes	Yes	Yes	Yes	Yes
R^2^	0.88	0.88	0.86	0.73	0.73	0.74

Note: The dependent variable in columns (1) to (3) is the log value of innovation vitality. The dependent variable in columns (4) to (6) is the log value of entrepreneurial vitality. t-values are reported in parentheses. *, **, and *** indicate significance at the 10%, 5%, and 1% levels, respectively.

## 5. Conclusion

Innovation and entrepreneurship are the driving force of economic development [69]. Previous studies have examined innovation and entrepreneurship separately. Innovation and entrepreneurship are closely related, so only by combining them can we better study local innovation and entrepreneurship vitality. In this paper, we examine the association of housing prices and the vitality of innovation and entrepreneurship with a city-level panel dataset from 2009 to 2019. In theory, there are two mechanisms underlying the impacts of rising house prices on VIE. One is to promote VIE through the wealth effect and the siphon effect, and the other is to suppress VIE by crowding out funds and innovative talents for innovation and entrepreneurship activities.

Our results suggest that rising housing prices increase VIE. This finding is quite robust after a series of robustness checks, including IV approach, alternative dentition of key variables, and spatial econometric models. We also examine the potential mechanisms that housing prices affect VIE, and our results suggest a partial mediating effect of consumption willingness and consumption ability. This study has several limitations, which imply the directions of future research. First, we summarize that housing prices affect VIE through crowding-out effect, wealth effect, and siphon effect. Future studies could use additional data to empirically verify these effects. Second, consumption willingness and consumption ability we identified only contribute to partial effects of housing prices on VIE. Other mediating variables are needed to explore.

Our results carry important policy implications. The positive relationship between housing prices and innovation and entrepreneurship documented in this paper suggests the critical role of the real estate market in economic development since innovation and entrepreneurship have long been considered driving forces of economic growth. When devising policies and regulations to promote innovation and entrepreneurship, policymakers should consider the role played by the housing market. We observe a promoting effect of housing prices on innovation and entrepreneurship, but high housing prices also have a crowding-out effect on innovation and entrepreneurship. It is necessary to curb the excessive rise of housing prices and reduce the absorption of capital and talent by the overheated development of real estate markets. In addition, decoupling public services and benefits related to homeownership can also reduce the possible negative consequences of high housing prices.

## Supporting information

S1 Data (XLS)
